# Supplementary material for: SpotLight Proteomics—A IgG-Enrichment Phenotype Profiling Approach with Clinical Implications
Source: Int J Mol Sci. 2019 May 1;20(9):2157. doi: 10.3390/ijms20092157 (PMC6540603; doi:10.3390/ijms20092157)
Supplement: Supplementary file 1 [file ijms-20-02157-s001.zip › Supplemental Figure S1.docx]

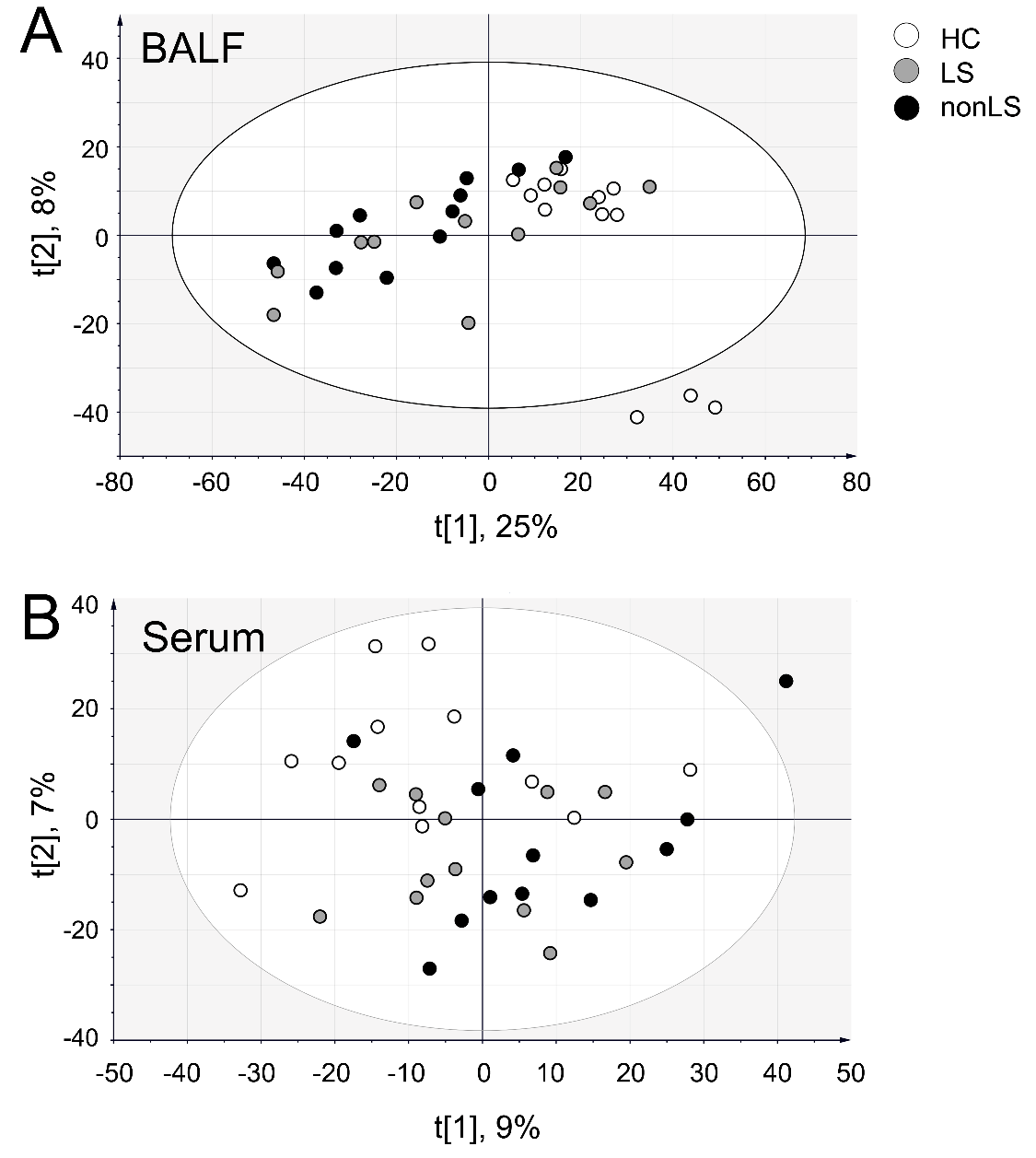


**Supplemental Figure S1.** PCA analyses of (**A**) the data acquired from the BALF samples and (**B**) the data acquired from the serum samples. Note that particularly the BALF- but also the serum samples cluster according to healthy controls (HC) and sarcoidosis (Löfgren’s Syndrome and non- Löfgren’s Syndrome; LS and nonLS).
